# Processing of Spontaneous Emotional Responses in Adolescents and Adults with Autism Spectrum Disorders: Effect of Stimulus Type

**DOI:** 10.1002/aur.1468

**Published:** 2015-03-03

**Authors:** Sarah Cassidy, Peter Mitchell, Peter Chapman, Danielle Ropar

**Affiliations:** ^1^ School of PsychologyUniversity of NottinghamScience ParkNottinghamNG7 2RD; ^2^ Centre for Research in Psychology, Behaviour and AchievementCoventry UniversityPriory StreetCoventryCV1 5FB

**Keywords:** autism spectrum disorders, face perception, eye tracking, spontaneous emotion recognition, retrodictive mindreading, social cognition, multi‐modal processing, visual auditory integration

## Abstract

Recent research has shown that adults with autism spectrum disorders (ASD) have difficulty interpreting others' emotional responses, in order to work out what actually happened to them. It is unclear what underlies this difficulty; important cues may be missed from fast paced dynamic stimuli, or spontaneous emotional responses may be too complex for those with ASD to successfully recognise. To explore these possibilities, 17 adolescents and adults with ASD and 17 neurotypical controls viewed 21 videos and pictures of peoples' emotional responses to gifts (chocolate, a handmade novelty or Monopoly money), then inferred what gift the person received and the emotion expressed by the person while eye movements were measured. Participants with ASD were significantly more accurate at distinguishing who received a chocolate or homemade gift from static (compared to dynamic) stimuli, but significantly less accurate when inferring who received Monopoly money from static (compared to dynamic) stimuli. Both groups made similar emotion attributions to each gift in both conditions (positive for chocolate, feigned positive for homemade and confused for Monopoly money). Participants with ASD only made marginally significantly fewer fixations to the eyes of the face, and face of the person than typical controls in both conditions. Results suggest adolescents and adults with ASD can distinguish subtle emotion cues for certain emotions (genuine from feigned positive) when given sufficient processing time, however, dynamic cues are informative for recognising emotion blends (e.g. smiling in confusion). This indicates difficulties processing complex emotion responses in ASD. ***Autism Res*** *2015, 8: 534–544*. © 2015 International Society for Autism Research, Wiley Periodicals, Inc.

## Introduction

Although current diagnostic criteria for autism spectrum disorders (ASD) includes difficulties interpreting other's emotions and responding appropriately (APA, 2013), emotion processing difficulties in ASD have not been consistently demonstrated (Gaigg, 2012; Harms, Martin, & Wallace, 2010; Uljarevic & Hamilton, 2013). Recent research suggests that the type of emotion expressions presented, and judgements made by participants in these studies do not match the demands of everyday life, where individuals with ASD are more likely to experience difficulties (Cassidy, Ropar, Mitchell, & Chapman, 2014). To understand the nature of the difficulties individuals with ASD experience, we must utilise tasks that match the demands of everyday life.

To accomplish this, Cassidy et al. (2014) developed a task to explore emotion processing in realistic social situations. Participants are presented with a person's spontaneous emotional response to a social situation (receiving a wanted or unwanted gift), and subsequently gauge the person's emotional response to infer what actually happened to them (what gift did they receive?). This ability has been termed Retrodictive Mindreading, and may be the most common form of emotion processing in everyday life (Millikan, 2005). Results showed that although adults with ASD understood what emotions were appropriate to each situation to the same extent as typical controls (e.g. feigning a positive response to an unwanted gift), they had difficulty interpreting subtle emotional responses, (genuine and feigned positive), but not confused, which was recognised to a similar level to typical controls.

It is unclear how individuals with ASD process the spontaneous emotional responses we typically encounter in everyday life. Spontaneous emotional responses are subtle, can show more than one emotion, and are subject to display rules, such as trying to show a positive, as opposed to a negative façade to a social interaction partner (Carroll & Russell, 1997; Matsumoto & Willingham, 2006; Matsumoto, Olide, Schug, Willingham, & Callan, 2009; O'Sullivan, 1982). Thus, spontaneous expressions have lower signal clarity than posed expressions, which tend to portray one emotion at a high intensity (Matsumoto et al., 2009). Not surprisingly, therefore, studies of typically developing adults have shown that spontaneous expressions are harder to recognise than posed expressions (Hoque & Picard, 2011; Hess & Blairy, 2001; Naab & Russell, 2007; Wagner, 1990; Wagner, Lewis, Ramsay, & Krediet, 1992; Wagner, MacDonald, & Manstead, 1986).

Studies of emotion processing in ASD have predominantly used posed expressions showing a single emotion (e.g. Adolphs, Sears, & Piven, 2001; Corden, Chilvers, & Skuse, 2008; Eack, Mazefsky, & Minshew, in press; Enticott et al., 2014; Ogai et al., 2003). These studies typically fail to find emotion processing difficulties in adolescents and adults with high functioning ASD (Gaigg 2012; Harms et al., 2010; Uljarevic & Hamilton, 2013), despite their difficulties interpreting emotions in everyday life (APA, 2013). This disparity between results in the lab and experience in the real world could be because the stimuli typically used (static posed expressions) have higher signal clarity than the spontaneous expressions encountered in everyday life. This theory is supported by studies showing that individuals with ASD have difficulties interpreting emotions of low intensity (Law Smith et al., 2010), blends of two emotions (Humphreys, Minshew, Leonard, & Behrmann, 2007), distinguishing genuine from feigned emotion responses (Boraston, Corden, Miles, Skuse, & Blakemore, 2008; Cassidy et al., 2014), and interpreting what happened to a person from their behaviour (Pillai et al., 2014). These studies suggest that adolescents and adults with ASD require a higher level of signal clarity than typical controls to successfully interpret emotions.

Another important component of emotional responses in everyday life is that they are dynamic, which individuals with ASD may have difficulty processing (Gepner & Feron, 2009). For example, children with ASD are better able to infer emotion from videos when slowed down (Gepner, Deruelle, & Grynfeltt, 2001; Tardif, Lainé, Rodriguez, & Gepner, 2007), and adults with ASD are better able to infer complex emotions (such as guilt) from static photos of the eye region of faces, than from a video of a social interaction (Roeyers, Buysse, Ponnet, & Pichal, 2001). However, adults with ASD only showed difficulty inferring certain prototypical emotions (i.e. sad) from dynamic (compared to static) stimuli and were significantly better at inferring anger from dynamic (compared to static) stimuli (Enticott et al., 2014).

Difficulties processing dynamic stimuli could be due to those with ASD missing pertinent social cues. For example, those with ASD show delays in fixating socially pertinent information in static pictures of social scenes, such as people and the eyes of faces (Fletcher‐Watson, Leekam, Benson, Frank, & Findlay, 2009; Fletcher‐Watson, Leekam, Findlay, & Stanton, 2008; Freeth, Ropar, Chapman, & Mitchell, 2010a, 2010b). This impacts processing of dynamic stimuli, which tends to reveal overall differences in visual fixation patterns in adolescents and adults with ASD (Klin, Jones, Schultz, Volkmar, & Cohen, 2002; Speer, Cook, McMahon, & Clark, 2007).

Although it appears that individuals with ASD have difficulty processing spontaneous emotional expressions due to their low signal clarity and fast paced dynamic cues, these factors have not been explored using spontaneous emotional responses. To explore these possibilities, we utilise the same task reported in Cassidy et al. (2014) under two conditions; the original videos of peoples' emotional responses, and static pictures of the peak of the expression presented for as long as participants need to judge the person's emotional response. If individuals with ASD primarily have difficulty interpreting others' emotional responses due to missing important cues from fast paced dynamic stimuli, then we would expect that freezing these cues for as long as participants needed would improve their ability to accurately gauge another's emotional response. However, if adolescents and adults with ASD primarily have difficulty interpreting others' emotional responses due to low signal clarity, then we would expect that freezing the expression for as long as participants need would not improve their ability to gauge others' emotional responses.

We also record participants' eye movements while viewing the stimuli, as eye‐tracking studies have shown reduced attention to social information particularly for dynamic stimuli (e.g. Speer et al., 2007). Thus, we explore whether individuals with ASD show reduced attention to the face of the person and eyes of the face more in the dynamic than the static condition, alongside increased difficulty interpreting dynamic emotional responses. If so, then this would further suggest that individuals with ASD have difficulty interpreting spontaneous emotional responses due to missing pertinent social cues from fast paced dynamic stimuli.

## Method

### Participants

The ASD group was comprised of 17 adolescents and adults (2 female, 15 male) aged 14–21 years, recruited from specialist schools and colleges for individuals with ASD across the UK. Two participants in the ASD group met the recommended cut‐off (>32) on the autism spectrum quotient (AQ) (Baron‐Cohen et al., 2001). However, all participants with ASD had been formally diagnosed by a clinician according to DSM‐IV criteria (American Psychological Association, 1994) before being accepted into the specialist school or college. The control group was comprised of 17 adolescents and adults (11 female, 6 male) aged 15–19 years, recruited from mainstream schools and colleges in the East Midlands area, without any diagnosed medical or learning difficulties. The full Weschler Abbreviated Subscales of Intelligence (WASI‐III) (Wechsler, 1999) was administered to all participants. Groups were matched on age and intelligence quotient (IQ; see Table 1), but not gender (*X* ^*2*^(1) = 10.1, *P* < 0.001). There was no significant effect of gender on task performance in the control group.

**Table 1:** Participant Characteristics

	ASD group (*N* = 17)	Control group (*N* = 17)	*t*‐test result
	Mean ± S.D. (range)	Mean ± S.D. (range)	
Age (years)	17.3 ± 1.6 (14–21)	17.1 ± 0.9 (15–19)	*t*(32) = 0.4, *P* = 0.69
Full‐scale IQ	92.2 ± 13.6 (74–120)	97.8 ± 6.4 (86–108)	*t*(32) = 1.5, *P* = 0.14
Verbal IQ	89.6 ± 15.2 (64–115)	96.3 ± 6.2 (85–106)	*t*(32) = 1.7, *P* = 0.11
Performance IQ	97 ± 13.5 (72–120)	99.2 ± 8 (86–111)	*t*(32) = 0.6, *P* = 0.58
AQ	22.8 ± 7.7 (11–39)	N/A	N/A

N.B. AQ score is missing for one participant with ASD.

### Materials

The dynamic condition included 21 video clips (ranging from 1.3 to 6 sec in duration). The static condition included 21 pictures taken from one frame of each video. Stimuli were presented on the Tobii (1750) eye‐tracker in high definition (1920 × 1080i). Eye movements were recorded using Tobii Studio at a rate of 50 recordings per second.

### Stimuli Development

#### Dynamic stimuli

Full details of the stimuli set are available in Cassidy et al. (2014). There were twenty‐one videos of people reacting to receiving a gift (a box of chocolates, a homemade novelty or a wad of Monopoly money) from the experimenter in exchange for staying behind after completing a long unrelated task. There were seven video‐taped reactions to each gift, which included verbal cues, varying in duration from 1.3 to 6 sec in length.

#### Static stimuli

One frame of each video clip was chosen by the researchers for use in the current study. This was judged by extracting the frame at the peak of the person's expression; after they had seen the gift, and their face had fully formed their reaction. Four independent judges rated whether the static picture chosen by the researcher represented the peak of the expression shown in the video. Cohen's Kappa showed a high level of agreement between judges (*K* = 0.86). These pictures were extracted in full colour high definition format using Final Cut Pro.

### Procedure

Participants took part in two sessions separated by a period of 2–4 weeks to reduce the possibility of carry‐over effects between the two conditions. In each session, participants were presented with video clips or static pictures while eye movements were recorded. The order of testing sessions was fully counterbalanced. Participants were given an information sheet about the study and asked to give their consent to take part. Participants sat in a comfortable chair approximately 40 cm from the eye‐tracking screen. A six‐point calibration was conducted before the start of the experiment and participants were asked to remain as still as possible throughout the experiment to prevent any deterioration in calibration.

Participants were told that they would see 21 videos or static pictures of people receiving either a box of chocolates, a wad of monopoly money or a hand‐made gift in exchange for doing a big favour for someone. They were asked to watch each video or picture carefully and judge what gift the target had been offered (of the three options), and state the emotion of the target on being offered the gift. All responses were verbal and digitally recorded.

In the dynamic condition, each trial presentation sequence consisted of a 500 ms blank screen preceding the video, followed by a fixation cross in the middle of the screen which participants were asked to fixate on while they gave their response. In the static condition, a fixation cross appeared in the middle of the screen for 5 sec before the picture appeared. The picture remained on the screen until the participant answered the test questions. Then, the experimenter would move onto the next trial by a key press. All pictures and videos were presented in random order.

The researcher then asked the participant ‘do you think the person got a box of chocolates, a tacky glitter card made especially for them, or some fake money?’ The participant was given as much time as they needed to respond to the test question. After giving a response the participant was then asked ‘How do you think the person felt when they got the [participant's gift response]?’ After the participant had responded to both of the test questions, the researcher asked if the participant was ready and started the next trial by a key press.

### Emotion Description Coding

As participants' estimations of the target's emotion were free response, to analyse these data the experimenter used the same coding scheme as described in Cassidy et al. (2014), to code participants' estimations of the recipient's emotion as belonging to one of four categories:

*Positive:* Any label which had a positive connotation; happy, glad, pleasantly surprised, pleased.

*Negative:* Any label which had a negative connotation; displeased, unhappy, disappointed, angry, upset.

*Pretend:* Any label which referred to the participant concealing negative emotions; hiding disappointment, fake smile, politely accepting.

*Confused:* Any label which did not have an explicit positive or negative connotation. For example, surprised, confused, puzzled, thoughtful.

## Results

### Behavioural Results

**Figure 1:**
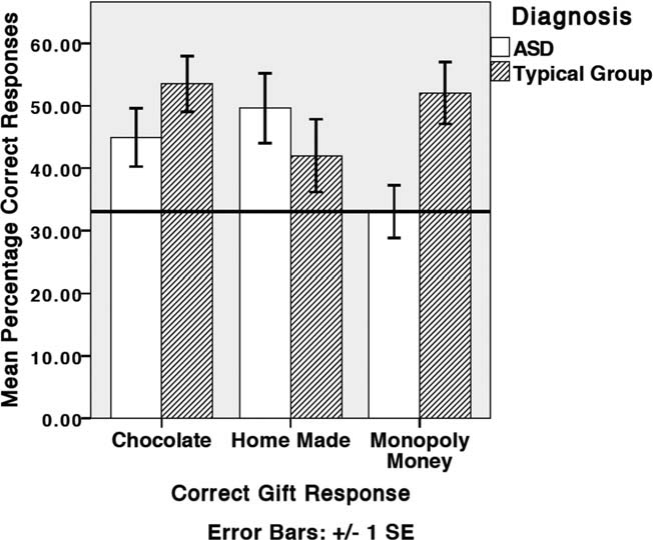
Percentage correct gift inferences in the ASD and typical group in the static condition. Horizontal line denotes chance level of 33%.

**Figure 2:**
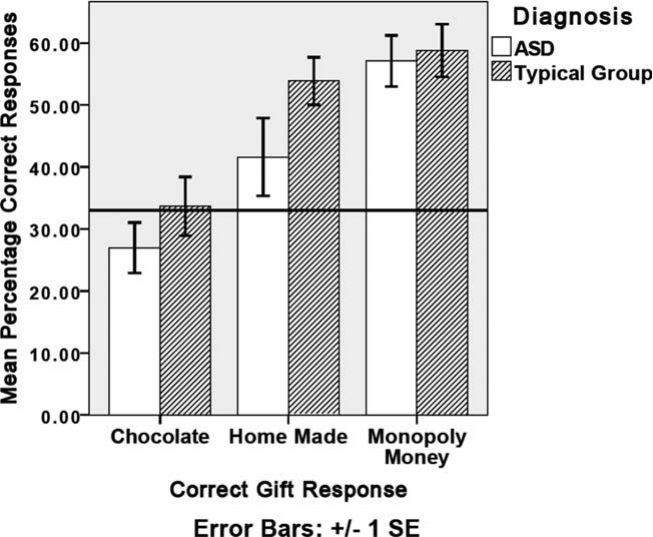
Percentage of correct gift inferences for the ASD and typical group in the dynamic condition. Horizontal line denotes chance level of 33%.

***Are individuals with ASD less accurate when inferring gift in both conditions?*** Table 2 shows the confusion matrices for participants' gift inferences in the typical control (a) and ASD (b) groups in each condition. In the dynamic condition, both groups perform comparably for chocolate, making more incorrect than correct inferences. Only typical controls made more correct than incorrect responses for a homemade gift, whereas both groups give more correct than incorrect responses for Monopoly money. In the static condition, both groups showed an increase in correct chocolate responses, and those with ASD an increase in correct homemade gift responses. However, those with ASD were less accurate than controls for Monopoly money. To control for ‘don't know’ responses, the proportion of correct gift responses were calculated as the number of correct responses, divided by the total number of times a participant offered that gift response (Figs. 1 and 2).

**Table 2:** Confusion Matrices Showing Raw Frequencies with which Each Gift was Inferred in Each Condition in the ASD and Typical Control Groups

		Dynamic Condition				Static Condition			
		Correct Answer				Correct Answer			
		Chocolate	Homemade	Monopoly money	Total	Chocolate	Homemade	Monopoly money	Total
a) Typical group									
Gift response	Chocolate	32_**A**_	44_**a**_	17_**b**_	93	54_**A**_	33_**a**_	19_**b**_	106
	Homemade	38_**a**_	63_**A**_	20_**b**_	121	37_**b**_	41_**A**_	34_**b**_	112
	Monopoly money	45_**a**_	6_**b**_	72_**A**_	123	20_**b**_	38_**a**_	59_**A**_	117
	Don't know	4	6	10	20	8	7	7	22
	Total	119	119	119		119	119	119	
b) ASD group									
Gift Response	Chocolate	33_**A**_	54_**a**_	25_**b**_	112	53_**A**_	28_**b**_	37_**a**_	118
	Homemade	46_**a**_	50_**A**_	28_**b**_	124	24_**b**_	58_**A**_	38_**a**_	120
	Monopoly money	38_**a**_	14_**b**_	66_**A**_	118	42_**a**_	30_**a**_	43_**A**_	115
	Don't know	2	1	0	3	0	3	1	4
	Total	119	119	119		119	119	119	

*Note*. Shaded cells denoted by **A** indicate correct gift inferences. Cells sharing a common subscript letter (**a**) were not significantly different from the correct gift response, while subscript letter (**b**) denotes a significant difference from the correct gift response.

A three way mixed ANOVA compared group (ASD, typical control), condition (static, dynamic) and percentage of correct gift responses (chocolate, homemade and Monopoly money). There was no significant effect of condition (*F*(1,32) = 0.3, *P* = 0.9) or group (*F*(1,32) = 2.4, *P* = 0.1). There was a three way interaction between group, condition and gift (*F*(2,64) = 7.2, *P* < 0.001). Simple main effects analysis showed participants with ASD were only significantly less accurate than controls when inferring who received Monopoly money in the static condition (*F*(1,32) = 8.5, *P* < 0.01).

#### Does performance improve in the static condition?

Simple main effects analysis showed participants with ASD were significantly more accurate when inferring who received a chocolate gift (*F*(1,32) = 8.1, *P* < 0.01), and significantly less accurate when inferring who received Monopoly money (*F*(1,32) = 18.1, *P* < 0.001) in the static (compared to the dynamic) condition. There was no significant difference in correct homemade gift inferences between the two conditions (*F*(1,32) = 2.1, *P* = 0.1).

The typical control group were significantly more accurate when inferring who received a chocolate gift (*F*(1,32) = 9.9, *P* < 0.01), and marginally significantly less accurate when inferring who received a homemade gift (*F*(1,32) = 0.8, *P* < 0.05) in the static (compared to the dynamic) condition. There was no significant difference in correct Monopoly money (*F*(1,32) = 1.4, *P* = 0.2) inferences between the two conditions.

A two way mixed ANOVA showed that the static picture was displayed longer in the ASD than the typical control group (*F*(1,32) = 9.8, *P* < 0.01), regardless of gift type (*F*(2,64) = 2, *P* = 0.14).

#### What was the pattern of errors in each group and condition?

Table 2 shows that participants with ASD confuse reactions to chocolate and homemade less in the static (compared to the dynamic) condition, and confuse reactions to Monopoly money with chocolate and homemade more in the static (compared to the dynamic) condition. To compare this pattern of errors between groups and across conditions, a four way mixed ANOVA was conducted with group as a between subjects factor with two levels (ASD, typical), condition as a within subjects factor with two levels (static, dynamic), correct answer (i.e. what gift the target received) as a within subjects factor with three levels (chocolate, homemade and Monopoly money) and participants' response as a within subjects factor with three levels (chocolate, homemade and Monopoly money). The four way mixed ANOVA showed a significant interaction between group, condition, correct answer and participants' gift response (*F*(4,128) = 4.1, *P* < 0.01). To explore this interaction, simple main effects analysis compared the percentage of correct to incorrect responses in each condition and group separately, followed up with Bonferroni corrected *t*‐tests.

### Dynamic Condition

Participants with ASD gave significantly more correct Monopoly money inferences than incorrect chocolate (*P* < 0.001) and homemade (*P* < 0.001) inferences (*F*(2,31) = 18.1, *P* < 0.001); significantly more correct homemade inferences than incorrect Monopoly money (*P* < 0.01), but not chocolate (*P* = 0.9) inferences (*F*(2,31) = 20.7, *P* < 0.001); participants with ASD did not give significantly more correct chocolate inferences than incorrect homemade or Monopoly money inferences (*F*(2,31) = 0.8, *P* = 0.5).

Typical controls gave significantly more correct Monopoly money inferences than incorrect chocolate (*P* < 0.001) and homemade (*P* < 0.001) inferences (*F*(2,31) = 26, *P* < 0.001); significantly more correct homemade inferences than incorrect Monopoly money (*P* < 0.001), but not chocolate (*P* = 1) inferences (*F*(2,31) = 39.3, *P* < 0.001); typical controls did not give significantly more correct chocolate inferences than incorrect homemade or Monopoly money inferences (*F*(2,31) = 0.4, *P* = 0.7).

### Static Condition

Participants with ASD did not give significantly more correct Monopoly money inferences than incorrect chocolate or homemade inferences (*F*(2,31) = 0.01, *P* = 0.9). Participants with ASD gave significantly more correct homemade inferences than incorrect chocolate inferences (*P* < 0.05), but not incorrect Monopoly money (*P* = 0.053) inferences (*F*(2,31) = 4.3, *P* < 0.05); and significantly more correct chocolate inferences than incorrect homemade inferences (*P* < 0.01), but not incorrect Monopoly money (*P* = 1) inferences (*F*(2,31) = 7.1, *P* < 0.01).

Typical controls gave significantly more correct Monopoly money inferences than incorrect chocolate (*P* < 0.001) and homemade (*P* < 0.01) inferences (*F*(2,31) = 11.1, *P* < 0.001); significantly more correct chocolate inferences than incorrect homemade (*P* < 0.05) and incorrect Monopoly money (*P* < 0.001) inferences (*F*(2,31) = 13.3, *P* < 0.001); but not significantly more correct homemade inferences than incorrect Monopoly money or chocolate inferences (*F*(2,31) = 1, *P* = 0.4).

### Summary

In the static (compared to the dynamic) condition, both groups made significantly more correct chocolate inferences, but individuals with ASD gave significantly less correct Monopoly money inferences. This is reflected in the pattern of participants' errors; participants with ASD could only distinguish reactions to chocolate and homemade in the static condition, and reactions to Monopoly money from chocolate and homemade in the dynamic condition.

#### Are gift and emotion inferences consistent?

A similar analysis approach to Cassidy et al. (2014) is adopted here. Likelihood ratios compare the observed and expected frequencies of emotion labels participants offered alongside their gift inference, for correct (3) and incorrect (4) trials in each group and condition. Tables 3 and 4 show that typical controls (3/4a) and those with ASD (3/4b) rate chocolate inferences as predominantly positive, Monopoly money as predominantly confused, and higher than expected. Inconsistent gift and emotion responses (e.g. positive for Monopoly money) are lower than expected. Pretend emotion ratings were rare, however, both groups ascribe this rating to homemade gift inferences above the level expected.

**Table 3:** Frequency of Emotion Ratings for Correct Gift Inferences in the ASD and Typical Group, in the Static and Dynamic Conditions

		Dynamic condition				Static condition			
		Correct gift response (expected frequencies in brackets)				Correct gift response (expected frequencies in brackets)			
		Chocolate	Homemade	Monopoly money	Total	Chocolate	Homemade	Monopoly money	Total
a) Typical group									
Emotion	Positive	30_**A**_ (14.75)	45 (29.05)	2 (33.2)	77	47_**A**_ (26.65)	22 (20.23)	7 (29.12)	76
	Pretend	0 (1.34)	6_**A**_ (2.64)	1 (3.02)	7	0 (1.05)	3_**A**_ (0.8)	0 (1.15)	3
	Confused	1 (12.65)	9 (24.9)	56_**A**_ (28.45)	66	3 (16.13)	10 (12.25)	33_**A**_ (17.62)	46
	Negative	1 (2.87)	1 (5.66)	13 (6.47)	15	0 (7.36)	3 (5.59)	18 (8.04)	21
	Don't know	0 (0.38)	2 (0.75)	0 (0.86)	2	4 (2.8)	3 (2.13)	1 (3.06)	8
	Total	32	63	72	167	54	41	59	154
b) ASD group									
Emotion	Positive	29_**A**_ (15.95)	32 (24.16)	11 (31.89)	72	50_**A**_ (28.91)	25 (31.64)	9 (23.45)	84
	Pretend	0 (2.21)	6_**A**_ (3.36)	4 (4.43)	10	0 (1.72)	4_**A**_ (1.88)	1 (1.4)	5
	Confused	4 (11.07)	7 (16.78)	39_**A**_ (22.15)	50	2 (15.14)	22 (16.57)	20_**A**_ (12.29)	44
	Negative	0 (2.88)	3 (4.36)	10 (5.76)	13	0 (5.16)	4 (5.65)	11 (4.19)	15
	Don't know	0 (0.89)	2 (1.34)	2 (1.77)	4	1 (2.06)	3 (2.26)	2 (1.67)	6
	Total	33	50	66	149	53	58	43	154

*Note*. Frequencies with subscript A denote correct gift and consistent emotion inference.

**Table 4:** Frequency of Emotion Ratings for Incorrect Gift Inferences in the ASD and Typical Group, in the Static and Dynamic Conditions

		Dynamic condition					Static Condition				
		Incorrect gift response (Expected frequencies in brackets)					Incorrect gift response (Expected frequencies in brackets)				
		Chocolate	Homemade	Monopoly money	Don't know	Total	Chocolate	Homemade	Monopoly money	Don't know	Total
a) Typical group											
Emotion	Positive	51_**A**_ (24.72)	21 (23.5)	5 (20.67)	0 (8.1)	77	46_**A**_ (26.38)	35 (36.02)	21 (29.43)	1 (11.16)	103
	Pretend	0 (1.28)	3_**A**_ (1.22)	1 (1.07)	0 (0.42)	4	0 (1.28)	5_**A**_ (1.75)	0 (1.43)	0 (0.54)	5
	Confused	8 (17.98)	24 (17.09)	24_**A**_ (15.03)	0 (5.89)	56	2 (12.29)	20 (16.79)	26_**A**_ (13.71)	0 (5.2)	48
	Negative	0 (9.63)	9 (9.16)	21 (8.05)	0 (3.16)	30	1 (5.89)	11 (8.04)	11 (6.57)	0 (2.49)	23
	Don't know	2 (7.38)	1 (7.02)	0 (6.17)	20 (2.42)	23	3 (6.15)	0 (8.39)	0 (6.86)	21 (2.6)	24
	Total	61	58	51	20	190	52	71	58	22	203
b) ASD group											
Emotion	Positive	68_**A**_ (43.68)	28 (40.91)	19 (28.75)	0 (1.66)	115	57_**A**_ (31.06)	21 (29.62)	19 (34.4)	0 (1.91)	97
	Pretend	0 (1.9)	3_**A**_ (1.78)	2 (1.25)	0 (0.07)	5	0 (1.6)	4_**A**_ (1.53)	1 (1.77)	0 (0.1)	5
	Confused	6 (17.09)	23 (16.01)	16_**A**_ (11.25)	0 (0.65)	45	7 (21.77)	26 (20.77)	34_**A**_ (24.12)	1 (1.34)	68
	Negative	2 (9.87)	12 (9.25)	12 (6.5)	0 (0.37)	26	0 (5.12)	6 (4.89)	10 (5.67)	0 (0.31)	16
	Don't know	3 (6.46)	8 (6.05)	3 (4.25)	3 (0.24)	17	1 (5.44)	5 (5.19)	8 (6.03)	3 (0.33)	17
	Total	79	74	52	3	208	65	62	72	20	203

*Note*. Frequencies with subscript A denote correct gift and consistent emotion inference.

Results showed that both groups gave significantly more consistent and less inconsistent gift and emotion inferences than predicted by the model, when the gift inferred was correct in the static (Typical group, Lχ^2^(1) = 17.6, *P* < 0.01; ASD group, Lχ^2^(1) = 12.1, *P* < 0.01) and dynamic condition (Typical group, Lχ^2^(1) = 24.4, *P* < 0.01; ASD group, Lχ^2^(1) = 13.77, *P* < 0.01), or when the gift inferred was incorrect in the static (Typical group, Lχ^2^(1) = 13.85, *P* < 0.01; ASD group, Lχ^2^(1) = 14.1, *P* < 0.01) and dynamic condition (Typical group, Lχ^2^(1) = 15.8, *P* < 0.01; ASD group, Lχ^2^(1) = 8.9, *P* < 0.05).

### Eye‐Tracking Results

#### Analysis

Tobii Studio was used to define regions of interest (ROIs) in the static condition as to the eyes, mouth and body. In the dynamic condition, fixations were visually coded as to the eyes, mouth or body by the experimenter. Perusal of the raw eye‐tracking data showed loss of calibration (indicated by fixations made outside of the eye‐tracking area) was more prevalent in the ASD group. To control for differences in calibration quality between groups, ratios were calculated for attention to the eyes of the face (eye:mouth ratio = eyes / eyes + mouth) and to the face of the person (face:person ratio = eyes + mouth / eyes + mouth + body). Higher values for the eye to mouth ratio denote a greater proportion of fixations/duration of fixations on the eyes of the face. Higher values for the face to person ratio denote a greater proportion of fixations/duration of fixations to the face of the person.

#### Visual fixation patterns in the static and dynamic conditions

A three way mixed ANOVA compared group as a between subjects factor with two levels (ASD, typical control), condition as a within subjects factor with two levels (static, dynamic) and ROI as another within subjects factor with two levels (eye to mouth ratio, face to body ratio), for the percentage number/duration of fixations. Data were collapsed across the three gifts as the pattern of results did not significantly differ between gift types (Table 5).

**Table 5:** Mean Percentage Fixation Count and Duration to the Eyes of the Mouth, and Face of the Person, for the ASD and Typical Group, in the Static and Dynamic Condition Conditions

	Dynamic condition		Static condition	
	Mean (SD)			
	Eye to mouth ratio	Face to person ratio	Eye to mouth ratio	Face to person ratio
a) Fixation count				
Typical group	48.95 (23.89)	96.98 (1.91)	45.54 (23.48)	93.34 (4.8)
ASD group	42.23 (25.39)	91.04 (8.43)	44.02 (15.41)	76 (19.97)
b) Fixation duration				
Typical group	47.9 (27.52)	97.64 (1.3)	42.13 (28.61)	97.31 (2.23)
ASD group	40.56 (28.4)	93.28 (8.72)	40.25 (19.39)	81.19 (20.4)

For percentage number of fixations there was a significant main effect of condition; percentage number of fixations to the eyes of the face and face of the person were significantly higher in the dynamic (69.8%) than the static (65%) condition (*F*(1,32) = 4.3, *P* = 0.046). There was a significant main effect of ROI; proportion of fixations were significantly higher to the face of the person (89.3%) than the eyes of the face (45.2%; *F*(1,32) = 208, *P* < 0.001). The interaction between condition, ROI and diagnosis failed to reach significance (*F*(1,32) = 3.2, *P* = 0.08); those with ASD tended to be less focused on the eyes of the face in the dynamic condition, and to the face of the person in the static condition (Table 5). There was a marginally significant effect of group; those with ASD were significantly less focused on the eyes of the face and face of the person than controls (ASD mean = 63.3%, control mean = 71.2%; *F*(1,32) = 3.8, *P* = 0.058).

For proportion of fixation duration, there was a significant main effect of ROI; proportion of fixation duration was significantly longer to the face of the person (92.3%) than the eyes of the face (42.7%; *F*(1,32) = 177, *P* < 0.001). The interaction between condition, ROI and diagnosis failed to reach significance (*F*(1,32) = 3, *P* = 0.09); those with ASD tended to spend less time looking to the eyes of the face in the dynamic condition, and to the face of the person in the static condition (Table 5). Those with ASD did not spend significantly less time looking to the eyes of the face, and the face of the person than controls (ASD mean = 63.8%, control mean = 71.2%; *F*(1,32) = 2.6, *P* = 0.1).

## Discussion

This study explored what underlies the emotion processing difficulties adolescents and adults with ASD experience in everyday life. Specifically, whether individuals with ASD miss important cues from fast paced dynamic stimuli, or alternatively, whether they have difficulty interpreting emotion responses of low signal clarity. Results showed that individuals with ASD were significantly better able to distinguish who received a chocolate or homemade gift when stimuli were static and participants had time to peruse the stimuli for as long as necessary, compared to when responses were dynamic and fast paced. Unexpectedly, participants with ASD were significantly less accurate when inferring who received Monopoly money in the static (compared to the dynamic) condition, and significantly less accurate than controls, despite viewing the pictures for significantly longer than typical controls. The pattern of errors reflects this significant change in performance between the two conditions. In the dynamic condition participants with ASD were more likely to confuse reactions to chocolate and homemade gifts but not Monopoly money, similar to the results found in Cassidy et al. (2014). However, in the static condition of the current study, participants with ASD were more likely to confuse reactions to Monopoly money with reactions to a chocolate or homemade gift.

Both groups systematically attributed different emotions to each gift in both conditions. This suggests (as found in Cassidy et al., 2014) that both groups understood what emotions were appropriate to each gift; genuine positive for chocolate, feigned positive for homemade and confused for Monopoly money. Hence, differences in the pattern of performance between conditions are most likely due to differences in ability to recognise different emotion responses from static and dynamic stimuli. In the ASD group, recognition of genuine and feigned positive emotion responses improves from static (relative to dynamic) stimuli, whereas recognition of confused emotion responses is significantly less accurate from static (relative to dynamic) stimuli.

An alternative explanation is that perhaps the individuals in the videos did not portray the expected emotions for gift. For example, in the case of the homemade gift, people may have felt genuinely positive in appreciation of the effort made. Furthermore, given that spontaneous emotions tend to be mixed (e.g. Matsumoto & Willingham, 2006), different emotions may have appeared sequentially throughout the video (e.g. initial confusion before a fake smile). This variability could explain why chocolate and homemade responses were confused more in the dynamic condition. Increased accuracy in the static condition could, therefore, have resulted from reducing this variability, by the researchers choosing a frame which best represented the emotion they expected to be consistent with each gift. Hence, perhaps differences in performance between conditions were not due to differences in ability to interpret emotion. Rather, the responses to each gift were significantly more consistent with the researcher's expectations in the static, than the dynamic condition.

If this were the case, then we would not expect emotion inferences to be associated with correctly judging what gift the person received. However, our results (also see Cassidy et al., 2014) show that when participants correctly gauge what gift a person received (e.g. homemade), they also systematically infer emotion (feigned positive, as opposed to positive or confused). The static stimuli chosen by the researchers were also endorsed by independent judges blind to what gift the person received. Hence, it is unlikely that the frame chosen was biased by expectations of the researchers, but rather a true representation of the peak of the emotion portrayed in the videos. Therefore, differences in performance across conditions in the ASD group, most likely reflect differences in ability to infer different emotions from static and dynamic stimuli.

Boraston et al. (2008) found that adults with ASD had difficulty distinguishing genuine from feigned smiles from static images, however, the images were displayed briefly. In this study, the images were displayed for as long as participants needed to respond. Eye‐tracking studies have shown that adolescents and adults with ASD show a delay, rather than an absence of looking to pertinent social information (Fletcher‐Watson et al., 2009, 2008; Freeth et al., 2010a, 2010b), which could particularly impact processing of dynamic stimuli (Speer et al., 2007). Studies using dynamic, as opposed to static stimuli also tend to show more consistent differences in emotion processing ability between individuals with and without ASD (Roeyers et al., 2001), and when dynamic stimuli is slowed, this improves emotion recognition performance in children with ASD (Gepner and Feron, 2009; Tardif et al., 2007). Our findings that individuals with ASD improve when interpreting genuine and feigned positive emotion responses from static (compared to dynamic) stimuli is consistent with these findings. This suggests that individuals with ASD can interpret subtle spontaneous emotion responses if given sufficient time to process visual cues. This interpretation is somewhat supported by our eye‐tracking data, which showed individuals with ASD looked (marginally) significantly less to the person than controls in both conditions, however, this group difference was not significant for duration of fixations. Those with ASD also tended to look less to the eyes of the face in the dynamic (and not the static) condition, and less to the face of the person in the static (and not the dynamic) condition than controls, but this interaction was not significant.

Freezing the emotion expressions significantly reduced ability to interpret confused emotion responses in the ASD group, suggesting that dynamic cues are necessary for those with ASD to interpret these responses. This is consistent with previous research showing that dynamic cues help adults with ASD recognise certain negative emotions such as anger (Enticott et al., 2014). Negative emotions (e.g. fear, anger) tend to reveal more consistent emotion recognition difficulties in ASD (Adolphs et al., 2001; Corden et al., 2008; Law Smith et al., 2010). This could be due to mixed cues from the mouth and eyes. Emotion blends (e.g. happy and surprised) also show subtle emotion recognition difficulties in ASD (Humphreys et al., 2007). People tend to show mixed emotions spontaneously, such as smiling in frustration (Hoque and Picard, 2011), and happily or angrily surprised (Du, Tao, and Martinez, 2014). These mixed emotional responses are characteristic of responses to Monopoly money, (e.g. smiling in confusion). Thus, the static images of spontaneous confused responses may not have had high enough signal clarity for individuals with ASD to recognise, even when given sufficient time to peruse these cues.

Another possibility for difficulties interpreting static confused responses in participants with ASD could be due to additional cues in the dynamic stimuli. For example saying “OK, what for?” in response to monopoly money, and “Thank you!” in response to a chocolate or homemade gift. Cassidy et al. (2014) suggested that individuals with ASD may rely more on speech content when successfully distinguishing confused responses from genuine and feigned positive responses, whereas distinguishing genuine from feigned positive responses requires integration of speech content with inconsistent visual cues (saying thank you with a fake smile). Previous research has shown that adults with ASD rely more on speech content, rather than integrating nonverbal cues when interpreting complex emotions from videos of social interactions (Golan, Baron‐Cohen, Hill, & Golan, 2006). Adults with ASD are also less accurate when distinguishing consistent from inconsistent facial and vocal emotions (O'Connor, 2007), and children with ASD are less susceptible to the McGurk effect, tending to report the vocally produced syllable (Bebko, Schroeder, & Weiss, 2014). This suggests difficulties integrating multimodal cues, instead relying on verbal cues. This could cause adults with ASD to misinterpret complex emotions with inconsistent facial and vocal cues.

In conclusion, this study is the first to explore what underlies the emotion processing difficulties adolescents and adults with ASD experience in everyday life. Results demonstrate that the nature of the stimuli significantly affects emotion processing in ASD. For dynamic stimuli, adults with ASD tend to rely on speech content, rather than integrating nonverbal cues. When this speech content is absent in static stimuli, adults with ASD are no longer able to interpret mixed emotional responses, such as smiling in confusion. This indicates difficulties interpreting emotions of low signal clarity; in the case of dynamic stimuli where visual and vocal cues may be inconsistent (saying thank you with a fake smile), or a static picture of a mixed emotional expression in the absence of informative speech content (smiling in confusion). However, adults with ASD can process subtle visual cues distinguishing genuine from feigned smiles if given time to peruse these. These subtle difficulties cannot be revealed using static stimuli. Future studies must explore what factors contribute to the emotion processing difficulties adults with ASD experience using real life social situations.
